# Radiocarbon dating uncertainty and the reliability of the PEWMA method of time-series analysis for research on long-term human-environment interaction

**DOI:** 10.1371/journal.pone.0191055

**Published:** 2018-01-19

**Authors:** W. Christopher Carleton, David Campbell, Mark Collard

**Affiliations:** 1 Department of Archaeology, Simon Fraser University,University Drive, Burnaby, British Columbia, Canada; 2 Department of Statistics and Actuarial Science, Simon Fraser University,University Drive, Burnaby, British Columbia, Canada; University at Buffalo—The State University of New York, UNITED STATES

## Abstract

Statistical time-series analysis has the potential to improve our understanding of human-environment interaction in deep time. However, radiocarbon dating—the most common chronometric technique in archaeological and palaeoenvironmental research—creates challenges for established statistical methods. The methods assume that observations in a time-series are precisely dated, but this assumption is often violated when calibrated radiocarbon dates are used because they usually have highly irregular uncertainties. As a result, it is unclear whether the methods can be reliably used on radiocarbon-dated time-series. With this in mind, we conducted a large simulation study to investigate the impact of chronological uncertainty on a potentially useful time-series method. The method is a type of regression involving a prediction algorithm called the Poisson Exponentially Weighted Moving Average (PEMWA). It is designed for use with count time-series data, which makes it applicable to a wide range of questions about human-environment interaction in deep time. Our simulations suggest that the PEWMA method can often correctly identify relationships between time-series despite chronological uncertainty. When two time-series are correlated with a coefficient of 0.25, the method is able to identify that relationship correctly 20–30% of the time, providing the time-series contain low noise levels. With correlations of around 0.5, it is capable of correctly identifying correlations despite chronological uncertainty more than 90% of the time. While further testing is desirable, these findings indicate that the method can be used to test hypotheses about long-term human-environment interaction with a reasonable degree of confidence.

## Introduction

Time-series regression analysis is an important tool for testing hypotheses about human-environment interaction over the long term. The primary sources of information about human behaviour and environmental conditions in deep time are the archaeological and palaeoenvironmental records, respectively. These records contain observations with an inherent temporal ordering and are thus *time-series*. This means time-series regression methods could be used to quantitatively test hypotheses about the impact of climate change on humans and other hominins, or conversely the impact of hominin societies on their environments. However, there is reason to think that chronological uncertainty may complicate the use of such methods. In particular, the chronological uncertainty associated with the most common chronometric method used in the dating of both records—radiocarbon dating—could undermine our ability to confidently identify statistical relationships between the records. This is because calibrated radiocarbon dates have highly irregular uncertainties associated with them, and uncertainties of this type are not in line with the assumptions of many standard statistical methods, including time-series analysis [1–5]. To investigate this possibility, we conducted a simulation study in which we investigated the impact of radiocarbon dating uncertainty on a time-series regression method that is well-suited for archaeological and palaeoenvironmental research—the Poisson Exponentially-Weighted Moving Average (PEWMA) method [6].

## Background

Time-series data have to be analyzed carefully because the order in the sequence of observations matters. There are two traits a time-series can have that make temporal ordering important. One is *non-stationarity*, which describes time-series with statistical properties that vary through time—e.g., the mean or variance of the series might change from one time to the next, violating the common statistical assumption that observations are identically distributed [7]. The other troublesome trait is *autocorrelation*, which means the observations in the series correlate with themselves at a given lag [7]. Autocorrelation leads to dependence among the observations in a time-series, which violates another common statistical assumption, namely that observations are independent.

Archaeological and palaeoenvironmental time-series typically have both traits [3,8,9]. They will usually be non-stationary, because almost all environmental or cultural phenomena change over time—e.g., yearly temperatures, or population demographics. They will also typically contain temporal autocorrelation. Thus, archaeological and palaeoenvironmental data can be expected to violate the assumptions of many statistical methods. Consequently, we need special methods to find correlations between past human and environmental conditions.

Fortunately, these methods already exist because statisticians, mathematicians, and engineers have been working with non-stationary, autocorrelated time-series for a long time [10]. As a result, many established time-series methods are designed specifically to handle non-stationary, autocorrelated data [7,8,11]. However, time-series of archaeological and palaeoenvironmental observations are idiosyncratic in another way that potentially undermines even these established methods—often we are uncertain about the precise times associated with the observations [12–14]. That is, the time-series contain *chronological uncertainty*.

Contemporary time-series, such as stock prices or daily temperatures, are usually recorded at precisely known times, but looking into the deep past entails significant chronological uncertainty. Archaeologists and palaeoenvironmental scientists usually make chronometric estimations by proxy using radiometric methods that rely on measuring isotopes of unstable elements that decay at a constant rate [15]. Even the most precise of these methods, however, yield uncertain dates, some with decadal error ranges and others with centennial or millennial error ranges. Consequently, many palaeoenvironmental and archaeological time-series contain temporal uncertainty.

The most common chronometric method, radiocarbon dating, is particularly problematic. Radiocarbon dates have to be calibrated to account for changes in isotope ratios through time. The calibration process results in chronometric errors that are often highly irregular, yielding ranges of potential dates spanning many decades or even centuries [4,5,16,17]. Point estimates—i.e., mean ages—cannot be used to describe these distributions because they often contain multiple modes and are highly skewed [4,5]. Most statistical methods are, therefore, undermined by calibrated radiocarbon dating because most methods rely, at least to some extent, on point estimates. Time-series methods are no different, raising concerns about our ability to use them for identifying correlations between archaeological and palaeoenvironmental time-series.

In the study reported here, we explored the impact of chronological uncertainty on a time-series regression method called the Poisson Exponentially Weighted Moving Average (PEWMA) method [6]. Classified as a state-space time-series method, the PEWMA method models physical and natural systems as a set of input and output variables. It can be thought of as a mathematical filter that takes input variables and produces outputs by estimating the relationships among the variables. As the name implies, the PEWMA algorithm estimates a regression model for Poisson processes—i.e., a process that produces a series of integer numbers. Importantly, the method accounts for autocorrelation and non-stationarity in the Poisson process. It is potentially useful for many archaeological and palaeoenvironmental applications because count data is common in these fields—e.g., counts of artifacts, sites, or first appearance dates of species in the fossil record.

Like other state-space models, the PEWMA model has two main parts. The first is called the *measurement equation*. Brandt et al. [6] define this as

$$p(y_{t}|\mu_{t})=\frac{\mu_{t}^{y_{t}}e^{-\mu }}{y_{t}!},$$

where

$$\mu_{t}=\mu_{t-1}^{*}e^{X_{t}\delta }$$

and

$$\mu_{t-1}^{*}∼\Gamma (a_{t-1},b_{t-1}).$$

The measurement equations represent the observed count data as outcomes of a sequence of Poisson random variables. Each observation, *y*, is dependent on the unobserved mean of the Poisson process, *μ*_*t*_, at time *t*. The unobserved mean of the Poisson process, *μ*_*t*_, is, in turn, dependent on the mean at the pervious time, $\mu_{t-1}^{*}$. The previous mean is not merely a lagged value, though, which is why the asterisk is used. Instead, it is specified by a Gamma distributed prior denoted in the third equation by Γ, which has two parameters *a*_*t-1*_ and *b*_*t-1*_ corresponding to the shape and rate of the distribution, respectively. The unobserved mean at time *t* is also dependent on the regression term *e*^*X*^_*t*_^*δ*^ where ^*X*^_*t*_ is a matrix of covariates and ^*δ*^ is a vector of regression coefficients that is estimated from the data.

The second part of the PEWMA state-space model is called the *transition equation*. Brandt et al. [6] define the transition equation as

$$\mu_{t}=e^{r_{t}}\mu_{t-1}^{*}\eta_{t},$$

where

$$\eta_{t}∼\beta (\omega a_{t-1},(1-\omega )a_{t-1}).$$

These equations characterize the change in the unobserved mean through time. The first equation defines the mean at a given time, and has three terms. The first of these, $e^{r_{t}}$, describes the base rate of the mean process and ensures that the mean is always positive, which is necessary for Poisson processes. The second term, $\mu_{t-1}^{*}$, is the mean at the previous time—though, as we stated above, it is specified by a Gamma distributed prior and not merely a lagged mean value. To be consistent with the measurement equations, we added an asterisk to the term, making it slightly different from Brandt et al.’s [6] notation. The third term, *η_t_*, describes the stochastic shift in the mean from one time to the next. This term is Beta distributed, denoted in the second equation by β. It is defined by the two standard Beta parameters and a weight, *ω*, that discounts earlier observations exponentially—hence the “Exponentially Weighted” part of the PEWMA acronym. The *ω* parameter accounts for autocorrelation in the PEWMA model, and is estimated from the data. The parameters that appear in the Gamma and Beta distributions are also estimated from the data. Brandt et al. [6] calculate these parameters using recursive equations for *a* and *b* and a maximum likelihood approach. Online R scripts for estimating PEWMA models have been provided by Brandt et al. [6] (www.utdallas.edu/~pbrandt/pests/pests.htm).

To the best of our knowledge, the PEWMA method has only been used to analyze past human-environment interaction in one study [18]. In that study, we tested the prominent hypothesis that climate change exacerbates conflict within and between human societies over the long term (e.g., [19,20]). To test the hypothesis, we compared a time-series of Classic Maya conflict levels to several palaeoenvironmental proxies. The time-series of interest was a historical record of conflict events inscribed into monuments along with Classic Maya Long Count calendar dates. The conflict events include mentions of violent attacks, captive taking, human sacrifices, deliberate destruction of monuments, and large coordinated attacks timed to coincide with astronomical events [21,22]. Classic Maya elites had these events inscribed on monuments like door lintels in temples, stairways on pyramids, and most importantly large stone stelae [23]. The inscriptions describing these events generally include the date of the event in question, information about the nature of the event—e.g., the ruler of Caracol, a major centre, “decapitates/attack holy Mutal ajaw [a divine king connected to Tikal, another major centre]”—and the names of the relevant polities. Though not necessarily indicative of warfare in the modern sense, changes in the number of these events throughout the Classic Period likely indicates changes in the overall level of conflict among polities [18]. To create a time-series of these events, we counted the number of conflicts per 25-year period from 350–900 CE. The size of the interval was chosen to be consistent with earlier research, but we explored changing the size of the interval in subsequent analyses and obtained results that were consistent with those yielded by the main analyses (see the supplementary material associated with [18]).

Using the PEWMA method, we compared the conflict record with five palaeoenvironmental records including two temperature and three rainfall proxies. The temperature proxies are sea surface temperature (SST) reconstructions for the summer and winter seasons in the Cariaco Basin [24]. These records show an increase in SST over the Classic Maya period that correlate with other circum-Caribbean records over the same period. They also positively correlate with air temperature readings in the central Maya region during the 20^th^ century (see the supplementary material associated with [18]). The rainfall proxies included a titanium concentration record from the Cariaco Basin [25], an oxygen isotope record from a speleothem in southern Belize [21], and the well-known sediment density record from Lake Chichancanab located in the center of the Yucatan Peninsula [26]. In contrast to previous research on Classic Maya conflict [21], we found that temperature was the only variable that correlated significantly with conflict levels. We found no evidence for an impact of rainfall. From this, we concluded that increases in temperature might have led to increases in conflict among the Classic Maya, an idea not previously explored in the scholarly literature pertaining to the Classic Maya.

As the foregoing study suggests, the PEWMA method has the potential to improve our understanding of past human-environment interaction. However, given the ubiquity of chronological uncertainty in archaeological and palaeoenvironmental time-series, there is a need to better understand how chronological uncertainty affects the method—especially radiocarbon dating uncertainty, which is highly irregular, as we explained earlier.

To explore the effect of chronological uncertainty on the PEWMA method, we carried out a series of simulation experiments. The experiments involved creating thousands of pairs of artificial palaeoclimatic and archaeological time-series with known relationships and then testing for those relationships with the PEWMA method. The regressions were set up with the synthetic archaeological time-series as the dependent variable and the synthetic palaeoenvironmental time-series as the independent variable. We used error-free dates for the artificial archaeological time-series so that we could limit the sources of error and see the effects more clearly. This analytical control also had the benefit of allowing us to compare the simulation results to our previous work on the Classic Maya because the dependent variable in that study was a historical record with little chronological uncertainty [18]. Thus, in the present study only the synthetic palaeoenvironmental time-series contained chronological uncertainty. Using a bootstrap approach [27], we resampled the set of synthetic calibrated radiocarbon dates used to date the palaeoenvironmental time-series thousands of times, running a separate PEWMA analysis each time. For each experiment we varied several parameters while keeping everything else constant. The parameters included the variance of the time-series, the number of synthetic radiocarbon dates, and the strength of the correlation between the artificial archaeological time-series and the synthetic palaeoenvironmental data. Varying these parameters allowed us to see how radiocarbon dating uncertainty in the palaeoenvironmental series affected our ability to find the known relationships between the time-series in each pair.

## Methods

Using the R statistical programming language [28], we ran a series of simulation experiments, each of which explored how a set of variables affected the outcome of a PEWMA regression analysis. To reiterate, the PEWMA algorithm is a special kind of time-series filter that can be used to model Poisson processes containing autocorrelation and non-stationarity [6]. Poisson processes produce integer count time-series [29], a very common type of time-series in archaeology, as noted earlier—e.g., counts of sites per century or counts of animal bones per stratigraphic layer and so on. To model an empirical time-series, the PEWMA algorithm uses an *observe-then-predict* mechanism, which as the phrase suggests involves first observing some data and then making a prediction based on that observation. It filters through a given count series one observation at a time, updating its predictions for the next time based on previous observations. It can account for autocorrelation in the count data by discounting the information from older observations as it filters through the series. More discounting implies less autocorrelation in the observed data because older values in the series have a lower impact on subsequent values. The algorithm can also be fed covariates to see whether they improve its predictions of the time-series of interest. To estimate the statistical parameters for a model, the algorithm uses maximum likelihood, which means we can use Akaike’s Information Criterion (AIC), a measure of information loss, to estimate the goodness of fit of a given model [30–32]. Models with a lower AIC involve less information loss, meaning they fit the observed time-series better. The AIC we used is formulated as

$$AIC=(\left(-2*L)+(2*k\right))*\frac{N}{N-k-1},$$

where *L* is the log-likelihood of the model, *k* is the number of covariates, and *N* is the number of observations in the time-series. This formula is a small sample size-corrected version of the AIC, which is generally appropriate for archaeological research given the small numbers of observations typical of archaeological time-series.

In the simulations, we aimed to determine how calibrated radiocarbon date uncertainty affects the PEWMA model. Specifically, we sought to investigate the impact of radiocarbon date uncertainty on the PEWMA method when it is used to identify correlations between a calendrically-dated archaeological time-series and a radiocarbon-dated palaeoenvironmental time-series. To do so, we ran a massive simulation. The simulation was broken down into experiments. Each experiment involved a set of fixed parameters that were the same for every experiment and a set of variable, or free, parameters that we wanted to investigate. Within each experiment, 1000 pairs of synthetic time-series were analyzed using the PEWMA algorithm. We refer to these as the *top-level pairs*. Each top-level pair was subjected to a chronological bootstrap—i.e., random sampling of the radiocarbon date distributions used to date the synthetic palaeoenvironmental time-series—which resulted in 2000 *sub-pairs* of time-series. Each sub-pair only differed from the others because different dates were used to create their age-depth models.

The experiments involved several steps. First, we created 1000 synthetic palaeoenvironmental time-series spanning a thousand-year period, from 12000 to 13000 calibrated years BP, a fixed parameter of the experiments. This slice of the curve was chosen because it has a moderate amount of chronological uncertainty relative to older and younger periods, meaning our results should be relevant to a wide range of archaeological research. We created the observations in each series using a linear function with a slope of 0.01, also a fixed parameter. This function was chosen to simulate an environmental process that increased gently over the 1000-year period of the series—i.e., a synthetic environmental signal. We then added autocorrelated random error with a fixed autocorrelation of 0.7, creating noise in the synthetic environmental signal. The autocorrelated noise was generated using an R function called *arima*.*sim*. This autocorrelated component caused the linear signal to increase and decrease in a nonlinear fashion, mirroring the kind of variation commonly seen in palaeoenvironmental time-series. In each experiment, we controlled the amount of noise by tuning the standard deviation of the arima.sim function. The standard deviation could vary freely among three values, namely 1, 0.1, and 0.01. Increasing the standard deviation increased the level of noise, thereby decreasing the *signal-to-noise* ratio of the synthetic palaeoenvironmental observations—i.e., the variance of the autocorrelated noise increased relative to the variance of the signal. We then dated the observations by selecting radiocarbon dates from the INTCAL-13 calibration curve from 12000–13000 BP [33]. There could be five, 15, or 25 dates evenly spaced along the calendrical time axis of the curve. This parameter was intended to help us determine whether having more dates improved regression results. To derive dates in radiocarbon time, we looked up the radiocarbon dates in the curve that corresponded to the calendrical dates, a process sometimes called *back-calibration*. These back-calibrated dates became the synthetic radiocarbon assays for the time-series. They stood in for the uncalibrated radiocarbon measurements that we might receive from a dating lab in a real investigation. We then set the error of the simulated radiocarbon dates to a standard deviation of ± 50 years, a fixed parameter corresponding to a common magnitude of error returned by dating labs. Setting these errors to a constant value was necessary to isolate the errors introduced by calibration—i.e., the irregular uncertainties we were interested in.

**Fig 1:**
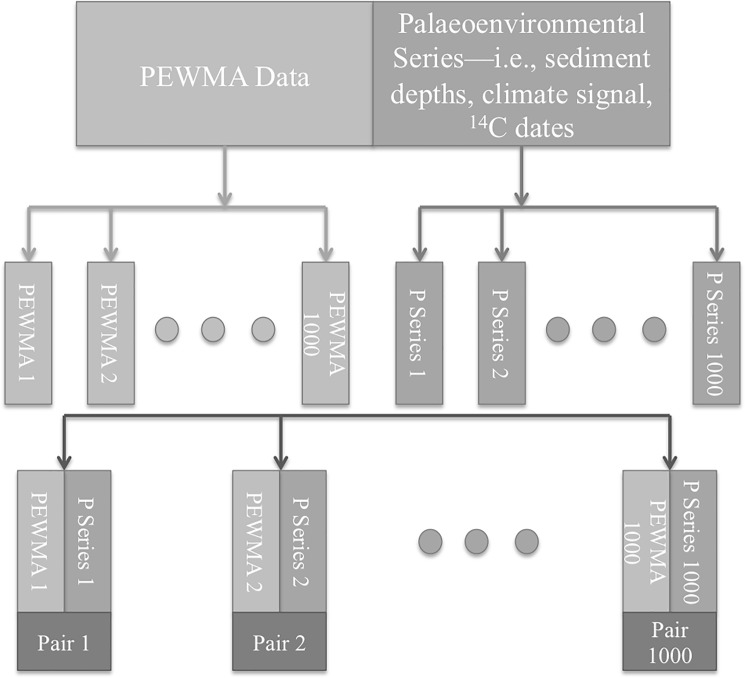
Flowchart showing the creation of the 1000 top-level time-series pairs, each comprising one simulated PEWMA series and one simulated palaeoenvironmental series.

In the second step, we created 1000 synthetic archaeological time-series using a PEWMA filter in reverse. Instead of iterating over an existing count time-series to estimate its statistical parameters, the algorithm can produce a time-series by feeding it a set of parameters. So, to simulate an archaeological process that was affected by environmental conditions, we fed in each of the synthetic environmental series created in the previous step. To do that, we sampled each 1000-year environmental series 200 times at regularly spaced intervals and used them as covariates in the creation of 1000 PEWMA count time-series, creating 1000 time-series pairs (see Fig 1). By tuning the correlation parameter, we could test whether the strength of the correlation between a given synthetic environmental time-series and its paired artificial archaeological time-series affected our results. To be clear, we were interested in how the strength of the underlying correlation affected our ability to identify the underlying relationship in the presence of chronological uncertainty. We were not trying to estimate its magnitude. The correlation parameter varied among 0.75, 0.5, 0.25, and 0—the last of these indicated no correlation, which allowed us to estimate the false positive error rate for the PEWMA method. The PEWMA filter also has an autocorrelation parameter, which indicates the degree of persistence in the underlying Poisson process—i.e., the degree to which future values are dependent on previous ones. We fixed this parameter at 0.6 for the simulation, corresponding to the default settings for *Pests*, the R software package written by the developer of the PEWMA method [6].

**Fig 2:**
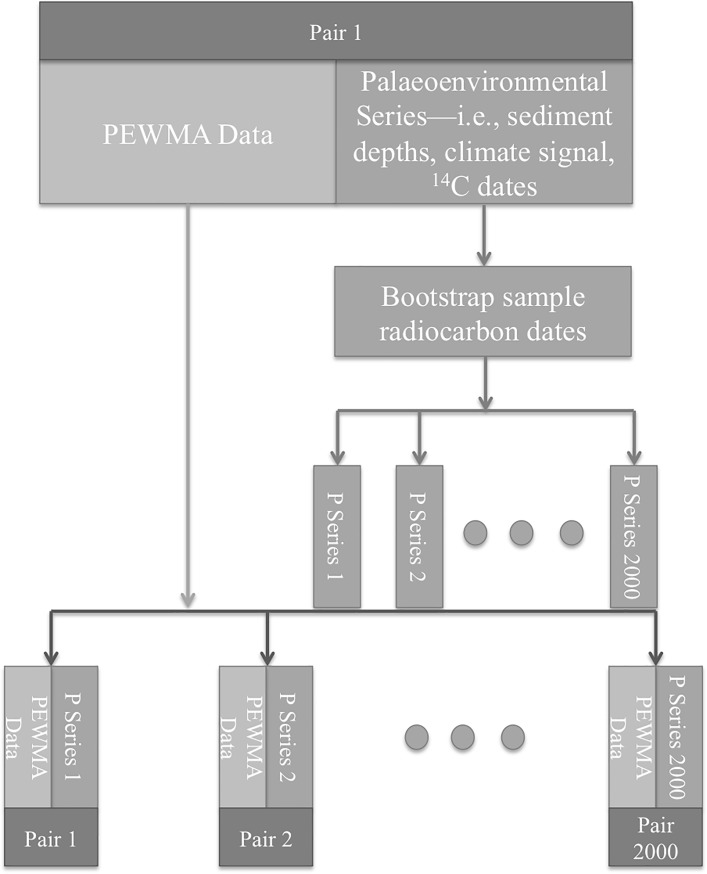
Flow chart showing the radiocarbon date bootstrap procedure and the creation of 2000 sub-pairs of time-series for the first pair of time-series created in the previous step. The same procedure was repeated for each of the 1000 top-level pairs at the bottom of Fig 1, resulting in a total of 2,000,000 simulated pairs of time-series for each experiment.

In the third step, we created 2000 age models for each of the 1000 synthetic environmental series (see Fig 2). Most palaeoenvironmental time-series are dated with age models—i.e., mathematical interpolations between chronometric estimates anchored to certain parts of a series [13,34]. The most common kind of age modeling involves sediment depths and radiocarbon dates. To date a time-series of observations from a lakebed sediment core, for example, palaeoenvironmental scientists interpolate between calibrated radiocarbon dates from a set of radiocarbon samples at different depths along the core. The depth of the carbon sample and its calibrated date become chronological anchors. By relating the age of the carbon sample to its depth, the ages of the layers between the anchors can be estimated. To simulate this process, while accounting for chronological uncertainty we used the bootstrap procedure, which is a method for estimating statistical parameters or distributions by random sampling with replacement [27]. The bootstrap involved calibrating the synthetic radiocarbon dates from the first step using R and then randomly sampling the calibrated distributions. We sampled them with replacement using a Gibbs sampler [16,35]—a tool that allowed us to randomly sample a sequence of radiocarbon dates with the constraint that the order of the dates in the time-series had to be preserved, mimicking stratigraphic relationships among them. Then, we used a monotonic spline to interpolate between the sampled radiocarbon dates, assigning a time stamp to each of the observations in a given synthetic environmental series.

In the last step of each experiment, we used the PEWMA method to create regression models with the synthetic archaeological time-series as dependent variables. For each archaeological time-series, we created 2000 PEWMA models. In each model, a given archaeological series was compared to one of the 2000 environmental series from its partner bootstrap ensemble. Since each of the 1000 archaeological time-series was paired to an ensemble of 2000 bootstrapped environmental time-series, we ran a total of 2,000,000 PEWMA analyses for each experiment. In each analysis, a given synthetic environmental time-series was used as a covariate for predicting its partner archaeological time-series. To determine whether including the environmental series improved a given model, we created another PEWMA model for each archaeological series that included only a constant and no covariate. The models with no environmental covariate acted as *benchmarks* for identifying statistically significant results. We reasoned that if the AIC of a given model with an environmental covariate outperformed its benchmark, the PEWMA algorithm had successfully identified the underlying correlation—or, in the case of no underlying correlation, erroneously identified one. For each of the 1000 synthetic archaeological series, we had 2000 PEWMA results, which meant we could calculate the percentage of the analyses that yielded a positive result—i.e., the *hit rate*. We then tallied these percentages to create a distribution of hit rates for each experiment.

## Results

**Fig 3:**
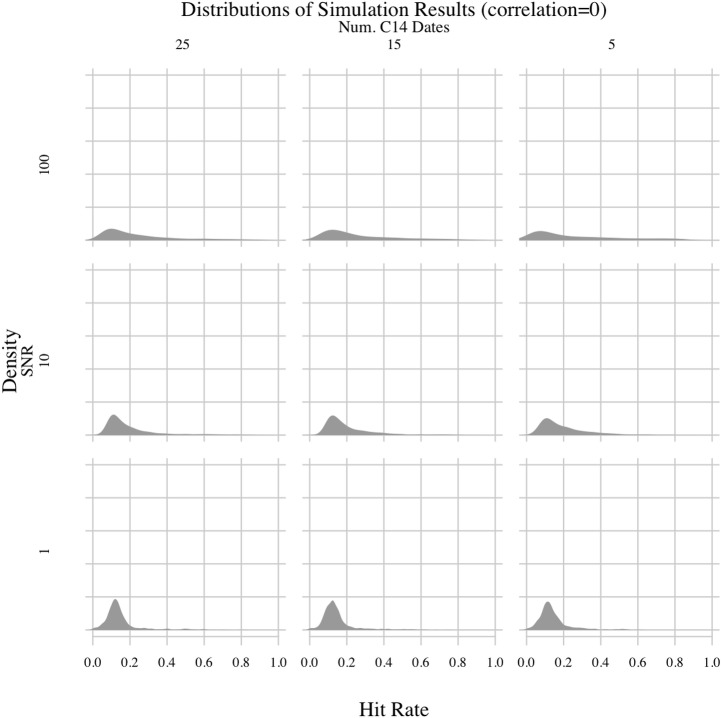
PEWMA simulation results; correlation = 0.

**Fig 4:**
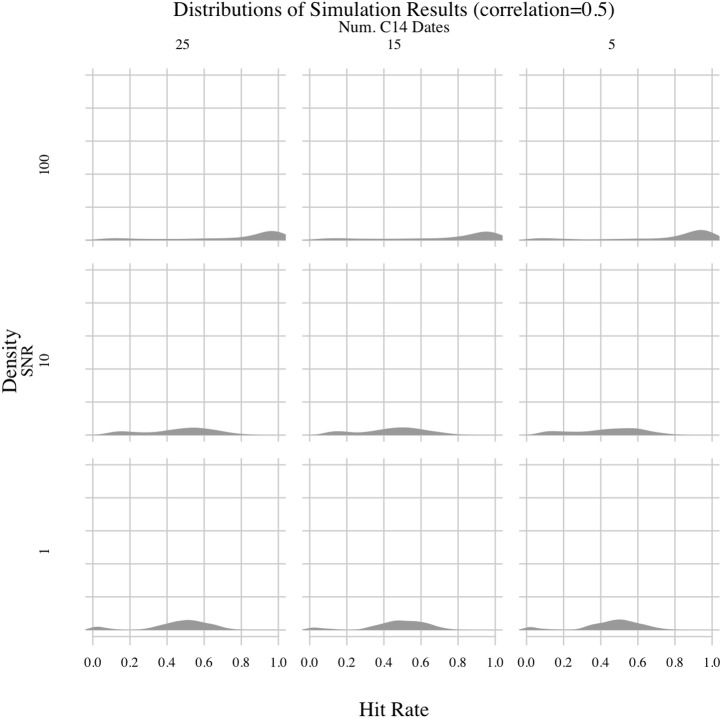
PEWMA simulation results; correlation = 0.25.

**Fig 5:**
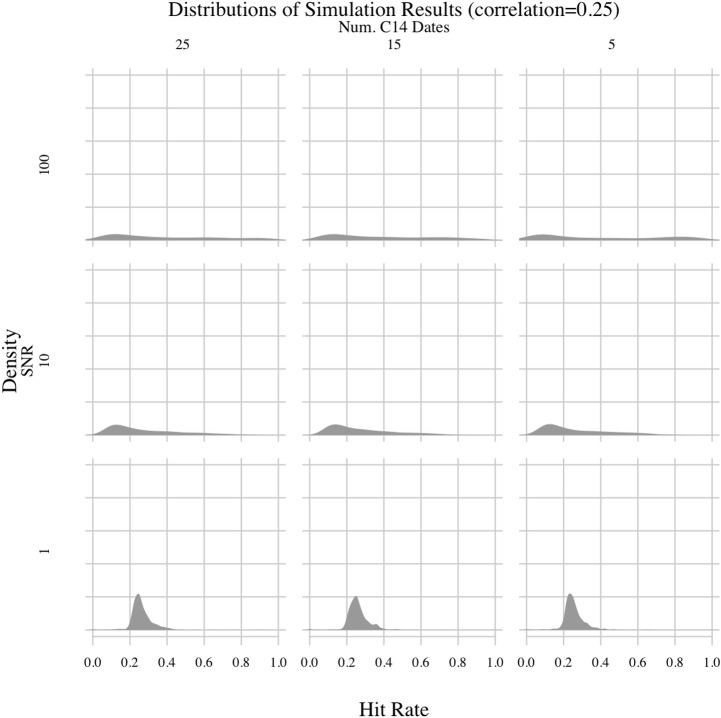
PEWMA simulation results; correlation = 0.5.

**Fig 6:**
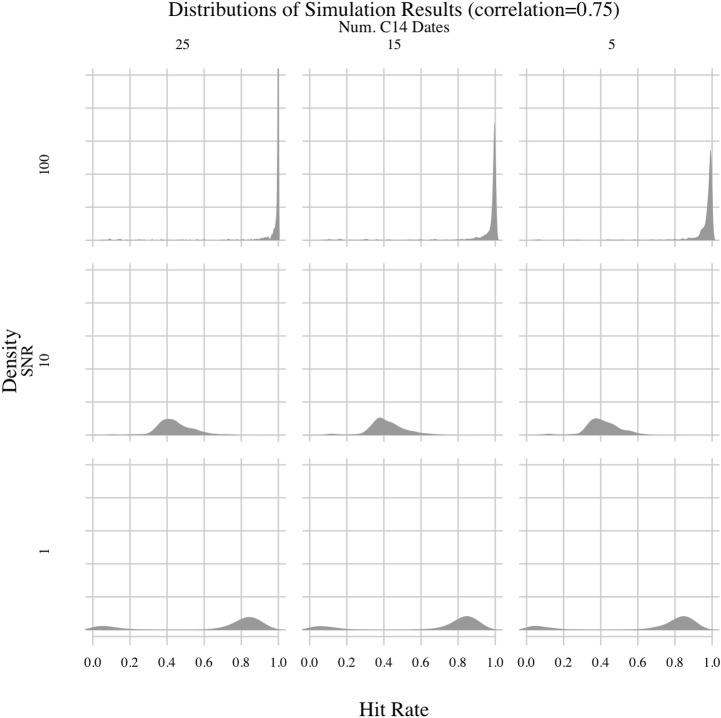
PEWMA simulation results; correlation = 0.75.

Permuting all possible values for the free parameters yielded 36 experiments, the results of which are shown in Figs 3–6. There are several important patterns in these results. The least surprising result involves the correlation between synthetic environmental and archaeological time-series. The correlation parameter had, by far, the clearest impact on hit rates. The method generally had a hit rate of less than 50% when the correlation was 0.25. Depending on the values of the other parameters, the hit rate varied between 20 and 40%. But, when the correlation increased to 0.5 or higher, the hit rate rose as high as 90% in experiments where the signal-to-noise ratio (SNR) was 100. As the correlation increased, the modes of the hit rate distributions increased and the variances generally decreased, meaning the method consistently performed better in experiments with higher correlations. Thus, when the environmental impact was greater, the PEWMA algorithm was better able to identify the underlying correlation despite radiocarbon dating uncertainty. This is an unsurprising finding because, intuitively, stronger relationships should be easier to identify.

Another unsurprising result involves the SNR. Holding the other parameters constant, we found that increasing the SNR from 10 to 100 generally improved the hit rate. When the SNR was 100, the PEWMA analysis was able to correctly identify the underlying correlation more than 80–90% of time in experiments with correlations of 0.5 or 0.75. Dropping the SNR to 10, though, reduced the hit rates. For the strongest correlation we explored—0.75—an SNR of 10 reduced the hit rate from greater than 80% to between 30% and 60%. For the lower correlation values, the hit rate was similarly reduced, but the distribution was also spread out across a greater range of values, indicating more variability in the hit rate as the SNR decreased. This finding makes sense since the climate data would be noisier, leading to a less clear relationship between the synthetic environmental series and the synthetic archaeological series.

Lowering the SNR further to 1 yielded what is, on the face of it, a counterintuitive result—the hit rate improved somewhat. For example, in experiments where the correlation was 0.75, reducing the SNR to 1 increased the mode of the hit rate distribution to more than 80%. This seems to suggest that noisier environmental data made it easier to identify an underlying correlation. However, the effect was caused by the fact that the autocorrelated noise we added to the main climate signal was included in the creation of the synthetic archaeological count data. So, increased environmental noise translated into increased noise in the archaeological data, too. Thus, when the correlation of a given experiment was strong, the increased variance of the environmental data resulted in higher overall co-variance of both time-series—both were noisy but strongly correlated. Consequently, the primary mode of the hit rate distribution shifted upward. Still, the hit rate distributions generally show higher variance as the SNR decreases, even in experiments with high correlations, which is more in line with the expectation that more noise should make it harder to see underlying relationships. In addition, a second mode appeared in the experiments with SNRs of 1 and correlations of 0.5 or 0.75. That smaller secondary mode in the hit rate distributions was much lower, around 10% or less. It indicates that the chances of failing to see the underlying correlation increased with very low SNR values, even in experiments with high correlations. Consequently, the overall effect of SNR values on the simulation was as expected, namely that more noise reduced the power of the method.

One surprising result involves the false positive rate of the PEWMA method. By setting the correlation of some experiments to zero, we were able to determine how often random variation resulted in spurious correlations. Overall, the modes of the hit rate distributions hovered around 10%, irrespective of the experimental parameters. Thus, the most common false positive hit rate for the PEWMA method appears to be around 10%. This false positive rate was lower than expected. Given the impact of radiocarbon dating uncertainty on other time-series methods we have explored (e.g., [3]), we were expecting to see more spurious correlations. So, a false-positive rate of about 10% seems very low and acceptable for archaeological applications. The hit rate distributions, however, are skewed to the right for experiments with higher SNRs, indicating greater numbers of spurious correlations. This finding makes sense considering those experiments involve synthetic environmental series with a straight, clearly increasing trend—i.e., strong signals with low noise. Holding that trend stable while allowing the synthetic archaeological series to fluctuate around it increased the chances that the two would align by chance. If, in contrast, the environmental series fluctuated more, we would expect to see fewer hits because chance concordances would occur less often. This is what we see. Decreasing the SNR led to noisier environmental series, which spuriously correlated with the synthetic archaeological series less often. Despite the difference caused by the SNR, though, the primary result is that the frequency of spurious correlations appears to have been low throughout the simulation, around 10%, even after accounting for radiocarbon dating uncertainty.

The last result is also surprising. It involves the number of radiocarbon dates. Surprisingly, increasing the number of radiocarbon dates used to date the time-series above five had little effect on the experimental hit rates compared to the other variables. Irrespective of the correlation and signal-to-noise ratios, the distributions of hit rates were almost identical whether the series were dated with five, 15, or 25 synthetic radiocarbon dates. So, from these results it appears that increasing the number of radiocarbon dates above five is unlikely to affect the accuracy of a PEWMA regression analysis even when using a bootstrap to account for dating uncertainty. This is surprising given our previous experience with radiocarbon dating uncertainty and its negative impact on time-series analyses. In a previous study [3], we determined that radiocarbon dating uncertainty undermined an established method for identifying cycles in time-series data. We found that radiocarbon dating errors led to the identification of spurious cycles in a drought proxy record from the Yucatan Peninsula, raising questions about the utility of time-series methods for identifying cycles in archaeological and palaeoenvironmental records. We anticipated similar findings for the present study, namely that radiocarbon dates would be a very important factor likely to undermine the method. Thus, it is both surprising and encouraging that varying the number of radiocarbon dates had little impact overall.

## Discussion

Our simulation experiments yielded three main findings regarding the impact of radiocarbon date uncertainty on the PEWMA method when it is used to identify correlations between a count-based archaeological time-series and a radiocarbon-dated palaeoenvironmental time-series:

1. The method’s true-positive rate ranges from 20–90%, with the most realistic rates being between 30 and 50%.
2. The method’s false positive error rate is approximately 10%.
3. Increasing the number of radiocarbon dates used to date the time-series above five had *no noticeable effect* on the true- or false-positive rates.

Taken together, the first two findings—a low false-positive rate and a moderate-to-high true-positive rate—indicate that the PEWMA method is suitable for research on past human-environment interaction. A low false-positive rate means we are reasonably unlikely to be fooled into thinking correlations exist when they do not—i.e., the method has a high *specificity*, a statistical term describing the rate of true-negative findings. A high specificity is ultimately the most important trait when investigating long-term human-environment interaction because spurious correlations abound in the real world and filtering out unlikely hypotheses is an important part of scientific research. On the other hand, the wide range of true-positive findings implies that we might miss important correlations because of chronological uncertainty, especially when the climate data are very noisy or the underlying correlation is weak. This is clearly a problem that should be addressed with more methodological work, but for now the PEWMA method appears to be a good tool for testing hypotheses involving correlations between palaeoenvironmental records and archaeological count data.

The third finding—that increasing the number of radiocarbon dates above five had no effect on the simulation results—is counterintuitive, though, and requires further thought. We initially expected that including more dates would markedly improve the true-positive rate and decrease the false positive-rate. That did not happen. One possible explanation for the counterintuitive relationship between dates and true-positive rates is that chronological uncertainty is not relevant at all because using more dates seemed to have no impact on the results. This possibility, however, can be dismissed by looking at the results of a single bootstrap iteration. Recall that the simulation was broken down into experiments. Each experiment involved a combination of simulation parameters that was constant throughout a given experiment. Within each experiment, 1000 pairs of synthetic time-series were analyzed using the PEWMA algorithm—the *top-level pairs*. Each top-level pair was subjected to a chronological bootstrap, which resulted in 2000 *sub-pairs* of time-series. Each sub-pair only differed from the others because different chronological anchors—i.e., dates sampled from calibrated radiocarbon date distributions—were used to create their age-depth models. So, if chronological uncertainty was irrelevant, we would expect the PEWMA analysis results to have been identical between sub-pairs. That is, we would expect that the PEWMA method would either succeed *or* fail 100% of the time for a given top-level pair because the sub-pairs only differed due to chronological uncertainty. What we saw instead was that each top-level result was a fraction ranging from zero to one, indicating the percentage of the 2000 sub-pairs for which the PEWMA method was able to identify the underlying correlation. Therefore, we can be sure that chronological uncertainty had an effect, which means that another explanation is required.

A more likely explanation, we think, is that chronological uncertainty has an effect, but it is not as important as the other variables, namely the signal-to-noise ratio and the strength of the underlying correlation. So, large differences in the signal-to-noise ratio and the strength of the underlying correlation will mask the effect of chronological uncertainty to some degree. Consequently, had we included chronological uncertainty in the archaeological time-series as well as the palaeoenvironmental time-series, we might have seen a greater effect. To some extent, therefore, these results should be considered relatively liberal, since archaeological time-series generally do contain chronological uncertainty. In a similar vein, had we used an older portion of the calibration curve or wider radiocarbon dating errors for the individual dates, we would expect the utility of the model to decrease. Still, since the effect we see in the simulation results is small, similar amounts of chronological uncertainty in the archaeological time-series, or small differences in other chronological uncertainties, should only slightly decrease the true-positive rate of the PEWMA method.

These findings have implications for our previous research on climate change and Classic Maya conflict [18]. As we explained earlier, the present simulation study compliments our earlier use of the PEWMA method for testing the hypothesis that climate change drove Classic Maya conflict. As part of our earlier research we performed sensitivity tests of the PEWMA method to account for various sources of bias. These tests indicated that our primary finding, that increases in temperature corresponded to increases in conflict at the centennial scale, was largely unaffected by temporal bias. However, it was a fairly limited evaluation of the PEWMA method. The present simulation looked specifically, and more completely, at the effect of chronological uncertainty in the palaeoenvironmental time-series by performing bootstraps to evaluate a very large number of what-if scenarios. The results suggest that the PEWMA method is robust to chronological uncertainty—in fact, chronological uncertainty appears to be the least important of the parameters we investigated. In addition, the portion of the calibration curve we used in the simulation is much older than the Classic Maya period, meaning it has greater chronological uncertainty associated with it. Even so, the simulation results suggest that false positive findings are rare. Importantly, the false positive rate would decrease for time-series spanning more recent periods because the chronological uncertainty in the calibration curve is lower over more recent periods as well. Thus, we can be more confident that our findings in the Classic Maya case study were not the result of chronological uncertainty.

To appreciate the implications of our simulation results more generally, we can think in terms of conducting *blind* analyses—i.e., real studies with no prior information about the existence, or non-existence, of an underlying relationship between human and environmental conditions. Imagine setting out to conduct a real analysis with the PEWMA method. Our simulation suggests that having at least five to 10 radiocarbon dates per 1000 years for a given palaeoenvironmental series is sufficient as long as those dates are spread fairly evenly throughout the series. Spending resources on more dates would likely make little difference in the results. This means, for instance, that most of the palaeoenvironmental time-series that are readily available online have sufficient numbers of radiocarbon dates to create reliable PEWMA models. The largest, and most popular, online source for palaeoenvironmental time-series is the NOAA website (www.noaa.gov). Perusal of their catalogue revealed that many of the time-series they curate come with more than five radiocarbon dates. Consequently, our hypothetical analysis could involve the existing palaeoenvironmental data, and if we need to gather a new dataset our chronometric costs would be low.

We could also be confident that our PEWMA analysis would be able to identify an important relationship if it existed, at least much of the time. Correlations with coefficients of 0.25 or greater were recoverable at least 20% of the time, and correlations of 0.5 or greater were recoverable upwards of 90% of the time. Thus, failing to find a relationship could suggest that there was no important relationship to find. If we hypothesized that rainfall variation, for instance, was strongly correlated to the rise and fall of Classic Maya socio-political complexity, then the PEWMA method should be able to identify such a relationship given a proxy time-series for past rainfall and one for socio-political complexity. If it failed to identify a relationship, one possible reason is that the correlation is quite low, at least according to our simulation results. Failing to find such a correlation, then, might simply indicate that the underlying relationship is not very important, falsifying the hypothesis that a strong relationship existed. On the other hand, for low to moderate correlations the method could miss a true relationship 50% of the time or more. A simple way to overcome this problem would be to test the hypothesis with additional time-series since that would increase the chances of finding a true-positive correlation. Therefore, with some replication we could be fairly confident in our findings.

It is important to keep in mind, though, that our simulations also imply that one in ten positive results might be spurious. There are at least two obvious ways to control for false positive findings. One is to use a more stringent test for statistical significance. Since the PEWMA method we used relies on comparing AICs to determine when a significant relationship has been identified, we could change the baseline for significance from identifying AICs that are strictly lower than a benchmark AIC to a baseline that required AICs to be lower by some predetermined amount, giving a confidence buffer of sorts. This is what we did in our previous analysis on climate change and Classic Maya conflict [18], and we strongly recommend it in general—though the specific size of the buffer is arbitrary and should be considered carefully for any specific case. The other way to control for false positives would be to conduct replication studies. For the hypothetical blind analysis we would have to gather multiple archaeological and palaeoenvironmental time-series containing observations of the same underlying phenomena—e.g., multiple proxies for Classic Maya socio-political complexity and multiple proxies for past rainfall. Then, we would re-run the PEWMA analysis and make a decision about our hypothesis on the basis of multiple results taken together, instead of relying on a single comparison. Overall, though, a false positive error rate of 1 in 10 seems acceptable for archaeological research. Therefore, while we ought to make attempts to control for the false positive findings, our simulation results suggest that the PEWMA method is adequate for archaeological purposes. It has a 90% chance of correctly determining that no relationship exists—i.e., a high specificity, as we mentioned earlier—if there is no underlying relationship and only a 10% chance of spuriously identifying one.

Overall, our results indicate that the PEWMA method is a promising time-series analysis tool for archaeological and palaeoenvironmental research. The method is suitable for analysing any archaeological count time-series, which potentially includes a wide range of archaeological proxies for past human behaviour, and it performs well even with relatively few radiocarbon dates—only five dates for a time-series 1000 years long. Therefore, we can make use of many of the published palaeoenvironmental time-series readily available online and maintain low chronometric costs when gathering new data. The method can also reliably find moderate to strong correlations between archaeological and palaeoenvironmental time-series when the latter have a strong signal. It should also be noted that leads and lags in a putative human-environment relationship could be tested for in the usual way—i.e., by included lagged versions of covariate time-series in the model. Thus, we think that the PEWMA method has the potential to contribute substantially to research on past human-environment interaction.

There is one very important caveat to keep in mind, which is that the results yielded by applications of the PEWMA method to archaeological time-series are assumption dependent. Like most statistical techniques, the PEWMA model was created with a specific class of problems in mind and therefore makes certain assumptions about the data. While it appears to be fairly robust to chronological uncertainty, it is best suited to cases where the count-based archaeological data represent a past process that 1) contained autocorrelation; 2) had temporal persistence that can be characterized adequately by exponential decay—e.g., the influence of past conflict levels or population sizes diminished exponentially with the passage of time; and 3) was subjected to temporally persistent effects from covariates. The last of these traits is particularly important because the PEWMA model assumes a given process was the product of its past states, which includes the previous impacts of any relevant covariates. So, the effect of covariates persists through time. If, in contrast, a process is suspected to have had covariates with only instantaneous impacts at any given time, then a PEWMA model may not be appropriate. It is, therefore, important to be aware of what one is attempting to model before using the PEWMA method. It would be wise to use the diagnostics outlined in Brandt et al. (2000) to determine whether a PEWMA model is suited to a given problem and dataset. It might also be useful to compare the PEWMA model results to other models, perhaps using AIC.

There are at least three avenues to explore in future research. One involves looking at the effect of calibrated radiocarbon date uncertainty on the dependent—i.e., response—variable. We chose to focus on chronological uncertainty in the palaeoenvironmental data in order to limit the sources of error in the simulation and see the effects of chronological uncertainty as clearly as possible. However, most archaeological time-series will likely contain chronological uncertainty, usually from radiocarbon dating. While we suspect the effect of additional radiocarbon dating uncertainty in the response time-series to be small—since the overall effect of chronological uncertainty appears to be small—it would still be prudent to investigate it further. Future research should involve simulations that look at how the PEWMA method performs when both the response and predictor time-series are dated with radiocarbon.

The second avenue for future research involves estimating the magnitude of an underlying correlation in the presence of chronological uncertainty. Our experiment involved determining whether we could identify an underlying correlation. An obvious parameter to explore, therefore, was the strength of that correlation, which we varied between experiments. The bootstrap simulations resulted in a range of estimates of the magnitude of correlations between the synthetic archaeological and palaeoenvironmental series. Clearly, it would be useful to use the bootstrap estimates to produce a single estimate for the underlying magnitude. That magnitude would indicate how important a given covariate was relative to other covariates, and it would also allow us to estimate effect sizes—i.e., the size of the impact that a given covariate had on the dependent archaeological time-series. However, combining the magnitude estimates from the chronological bootstrap is not straightforward and would have been an *ad hoc* exercise. In the future, we need to determine how best to combine the estimates while ensuring that the confidence intervals are calculated correctly. This research will require statistical development and further simulation work.

Lastly, it would be helpful to explore the impact of changing temporal scales on the PEWMA method. In the study reported here, we effectively used an annual resolution for the time-series, but often archaeological and palaeoenvironmental data have different resolutions. Many modern palaeoenvironmental records boast annual resolutions, for example, while most archaeological time-series will have much coarser resolutions. Consequently, we have to change the resolution of one or both time-series in order to perform analyses. Future research, therefore, should explore the effect of changing the resolutions of the independent and dependent time-series to match each other. Exploring these two potential research avenues would help us to determine the limits of the PEWMA method, a method with considerable potential to deepen our insights into past human-environment interaction.

## Conclusions

Time-series analysis has considerable potential to improve our understanding of past human-environment interaction. However, there is reason to think that its application could be undermined by the widespread reliance on calibrated radiocarbon dates for age-depth models. Calibrated radiocarbon dates have highly irregular uncertainties, as we mentioned earlier. These highly irregular uncertainties potentially pose a significant problem because they undermine the assumptions of standard statistical methods. With this in mind, we conducted a large simulation study in which we explored the effect of calibrated radiocarbon date uncertainty on a potentially useful Poisson regression-based method for time-series regression, called PEWMA. To test the effect of calibrated radiocarbon date error on the PEWMA method, we simulated thousands of archaeological and palaeoenvironmental time-series with known correlations and then analysed them with the PEWMA algorithm.

Our simulation experiments yielded three important findings. One is that the PEWMA method was able to identify true underlying correlations between the synthetic time-series much of the time. The true-positive rate for the method ranged from 20–90%, with higher true-positive rates when the synthetic environmental series contained less noise and the correlation between the time-series was stronger. Under the most realistic conditions, with moderate noise levels and correlation strengths, the true positive rate was around 30–50%. Decreasing the noise levels and increasing the correlation coefficients to 0.5 or 0.75 led to true positive rates upwards of 90%. While it is not surprising that stronger correlations in less-noisy data were easier to identify, it is important to be aware that the method might miss low correlation relationships.

The second important finding is that the false positive error rate of the method is roughly 10%, on average. This is surprising because we were expecting the highly irregular chronological errors of radiocarbon dates to warp the time-series in ways that could cause many spurious correlations and therefore a high false positive rate. Instead, the 10% false-positive rate suggests that finding spurious correlations is actually unlikely—in the context of archaeological research at any rate.

The third, and perhaps most surprising finding, was that varying the number of radiocarbon dates used to date the time-series had no noticeable effect. The true-positive rates were largely consistent whether five, 10, or 15 radiocarbon dates were used. This was surprising because it seems like adding more dates should reduce chronological uncertainty by increasing the number of chronological anchors for the age-depth models. Thus, we expected that more dates would improve our ability to find underlying correlations. That increasing the number of dates above five had no substantial impact on the true- or false-positive rates indicates that the PEWMA method is fairly robust to chronological uncertainty.

Taken together, our findings indicate that the PEWMA method is a useful quantitative tool for testing hypotheses about past human-environment dynamics. It can be used to determine whether an underlying correlation exists between a calendrically-dated archaeological time-series and a radiocarbon-dated palaeoenvironmental time-series. Crucially, it has a low false-positive rate, a moderate-to-high true-positive rate, and it appears to be fairly robust to chronological uncertainty. Methods with these traits are essential for analyzing archaeological and palaeoenvironmental time-series, which is a vital part of understanding past human-environment interaction.

## Supporting information

S1 File chrono.R. R script for building age-depth models.
(R) Click here for additional data file.

S2 File c14Bayes.R. R script for calibrating radiocarbon dates.
(R) Click here for additional data file.

S3 File simulation_pewma.R. R script containing low-level simulation functions.
(R) Click here for additional data file.

S4 File PEWMA_sim.R. R script with top-level simulation function—called from S5 File.
(R) Click here for additional data file.

S5 File hpc_pewma_exp1.R. R script for running the simulation in parallel.
(R) Click here for additional data file.
